# Crystal Crosslinked Gels with Aggregation-Induced Emissive Crosslinker Exhibiting Swelling Degree-Dependent Photoluminescence

**DOI:** 10.3390/polym9010019

**Published:** 2017-01-06

**Authors:** Tsuyoshi Oura, Ryosuke Taniguchi, Kenta Kokado, Kazuki Sada

**Affiliations:** 1 Graduate School of Chemical Sciences and Engineering, Hokkaido University, Kita10 Nishi8, Kita-ku, Sapporo 060-0810, Japan; yahoo@eis.hokudai.ac.jp (T.O.); r-taniguchi@frontier.hokudai.ac.jp (R.T.); 2 Department of Chemistry, Faculty of Science, Hokkaido University, Kita10 Nishi8, Kita-ku, Sapporo 060-0810, Japan

**Keywords:** aggregation-induced emission (AIE), crystal crosslinking, metal-organic framework, polymer gel

## Abstract

The synthesis and photoluminescence properties of crystal crosslinked gels (CCGs) with an aggregation-induced emission (AIE) active crosslinker derived from tetraphenylethene (TPE) is discussed in this article. The CCG was prepared from a metal organic framework (MOF) with large pore aperture to allow the penetration of TPE crosslinker. The obtained CCG possessed a rectangular shape originated from the parent MOF, KUMOF. The CCG showed stimuli-responsive photoluminescence behavior depending on the swelling degree, thus the photoluminescence intensity was higher at higher swelling degree. By changing the solvent, water content, or ionic strength, the photoluminescence intensity was controllable, accompanying the change of swelling degree. Moreover, emission color tuning was also achieved by the introduction of luminescent rare earth ions to form a coordination bonding with residual carboxylate inside the CCG.

## 1. Introduction

Aggregation-induced emission (AIE) dyes have recently become a new class of functional luminescent materials, because of their useful and attractive property wherein strong luminescence is observed in aggregated or solid states, while no or weak luminescence is observed in the dissolved state [1,2,3,4,5,6]. Compared to conventional luminescent dyes exhibiting concentration quenching, this property of AIE dyes offers great potential for practical use in fluorescent sensors [7,8,9], biological probes [10,11,12], or light emitting devices [13,14,15]. The restriction of intramolecular motion (RIM) process of densely packed aromatic rings is widely accepted to be a predominant cause of the AIE property.

The most common way to express the AIE property is to form an aggregation in a poor solvent via a reprecipitation process. However, aggregate formation is not always necessary to express AIE, and we can deduce that aggregate formation is not a requirement for AIE, but rather a sufficient condition. Therefore, other ways of providing the RIM process can also trigger AIE, other than aggregate formation. Recently, this idea has been realized by designing supramolecular interaction to suppress the molecular motion of AIE dye [16,17,18]. Shinkai et al. reported cyclization-induced emission driven by complexation between dicarboxylic acid and a tetraphenylethene (TPE) derivative, tethering zinc-dipicolylamine groups in homogeneous buffer solution [16]. Song et al. reported the occurrence of monomeric fluorescence via complexation between a TPE derivative and γ-cyclodextrin (γ-CD) in homogeneous solution [17]. Wu et al. reported fluorescence emergence induced by anion coordination of a TPE derivative having bisurea moieties [18].

As the environment to facilitate the RIM process of an AIE dye without aggregate formation, we focused on the use of a polymer gel and an elastomer consisting of network polymers crosslinked by an AIE dye [19,20,21,22]. Therein, the emission from AIE dye is feasibly controlled by interaction of the randomly-dispersed crosslinking point and the polymer chain, resulting in stimuli-responsive emission behavior of AIE dye toward swelling degree, salt concentration, solvent, temperature, or rigidity. As an unexplored network polymer matrix to control AIE, we conceived of the use of a crystal crosslinked gel (CCG), which is prepared via crystal crosslinking (CC)—a crosslinking reaction between porous crystal and an included crosslinker [23,24]. The method relies on the post synthetic method (PSM) of metal–organic frameworks [25,26], and the obtained crystal crosslinking gels possessed polyhedral shape derived from the original MOF crystals. Although CCG showed no apparent diffraction peaks on X-ray diffraction measurement, it should have higher molecular arrangement than conventional polymer gels, as illustrated its polyhedral shape. In this article, a TPE crosslinker suitable for the CC method was synthesized, and the environment-responsive AIE property was investigated. Our idea introduced herein has the potential to become a new strategy for the incorporation of dyes in polymer gels [27].

## 2. Materials and Methods

**Figure 1:**
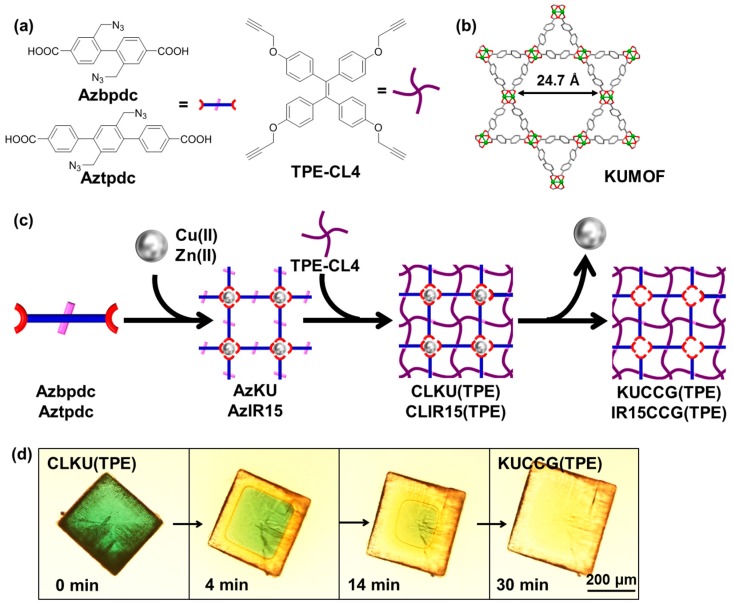
(**a**) Chemical structures of organic ligands, azide-functionalized biphenyl dicarboxylic acid (Azbpdc) and azide-functionalized terphenyl dicarboxylic acid (Aztpdc), and TPE-derived tetrapropargyl crosslinker (TPE-CL4); (**b**) Pore aperture of KUMOF from single crystal data [30]; (**c**) Schematic representation of crystal crosslinking method to obtain crystal crosslinked gel from azide-functionalized KUMOF (AzKU) (KUCCG(TPE)) and from azide-functionalized IRMOF-15 (AzIR15) (IR15CCG(TPE)) via crosslinked AzKU and AzIR15 with TPE-CL4 denoted as CLKU(TPE) and CLIR15(TPE); (**d**) Time-course observation of hydrolysis of CLKU(TPE) to obtain KUCCG(TPE) in conc. HCl/DMF = 1/5 (*v*/*v*).

The azide-functionalized biphenyl dicarboxylic acid (Azbpdc) [28], azide-functionalized terphenyl dicarboxylic acid (Aztpdc) [25], and TPE-derived tetrapropargyl crosslinker (TPE-CL4) [29] (Figure 1) were synthesized via the procedures outlined in the associated literature. Other reagents and solvents were purchased from commercial sources and used without further purification. All experiments were carried out under ambient atmosphere, unless mentioned. ^1^H NMR spectra were measured on a Bruker (Billerica, MA, USA) AVANCE DRX500 apparatus, using 0.05% tetramethylsilane (TMS) as an internal standard. Attenuated total reflection infrared (ATR-IR) spectra were obtained on a JASCO (Tokyo, Japan) FT/IR-4100 spectrometer with a diamond prism kit. X-ray diffraction (XRD) patterns were obtained by using a Bruker (Billerica, MA, USA) D8Advance with Cu Kα radiation source (40 kV, 40 mA). Optical/Polarization micrographs were obtained by using a Nikon instruments (Tokyo, Japan) SNZ1000 stereoscopic zoom microscope. Emission spectra were obtained with a Shimadzu (Kyoto, Japan) RF5300PC spectrofluorometer. The absolute photoluminescence quantum yield (Φ_F_) was measured by a Hamamatsu (Shizuoka, Japan) C9920-02 absolute photoluminescence quantum yield measurement system equipped with an integrating sphere apparatus and a 150 W continuous-wave xenon light source.

### 2.1. Preparation of AzKU (Azide-Functionalized KUMOF)

Azbpdc (25 mg, 0.071 mmol) and Cu(NO_3_)_2_·3H_2_O (25 mg, 0.10 mmol) were dissolved in 5 mL of *N*,*N*-diethylformamide (DEF) in a screw vial, and 0.1 mL DMSO was added to the solution. The vial was kept standing at 80 °C for 4 days. The solution was decanted, and green rectangular crystals were repeatedly washed with DEF.

### 2.2. Preparation of CLKU(TPE) (Crosslinked KUMOF with TPE-CL4)

AzKU was immersed in 5 mL of crosslinker solution of TPE-CL4 (0.1 M) in DEF and 25 μL of saturated Cu(I)Br solution of DEF, and stand for 5 days at 80 °C. The supernatant was decanted, and moss green rectangular crystals were repeatedly washed with DEF.

### 2.3. Preparation of KUCCG(TPE) (Crystal Crosslinked Gel from KUMOF)

CLKU(TPE) was immersed in a mixed solvent of conc. HCl/DMF (1/5, *v*/*v*) in a screw vial. The vial was kept standing at room temperature for 3 h. The supernatant was decanted, and brown cubic crystals were repeatedly washed with DEF after drying in an oven at 40 °C for 5 h.

### 2.4. Preparation of AzIR15 (Azide-Functionalized IRMOF-15)

Aztpdc (28 mg, 0.065 mmol) and Zn(NO_3_)_2_ 6H_2_O (57 mg, 0.19 mmol) were dissolved in 5 mL of DEF in a screw vial. The vial was kept standing at 80 °C for 3 days. The solution was decanted, and yellow cubic crystals were repeatedly washed with DEF.

### 2.5. Preparation of CLIR15(TPE) (Crosslinked IRMOF15 with TPE-CL4)

AzIR15 was immersed in 5 mL of crosslinker solution of TPE-CL4 (0.1 M) in DEF and 25 μL of saturated Cu(I)Br solution of DEF, and left for 5 days at 80 °C. The supernatant was decanted, and yellow cubic crystals were repeatedly washed with DEF.

## 3. Results

**Figure 2:**
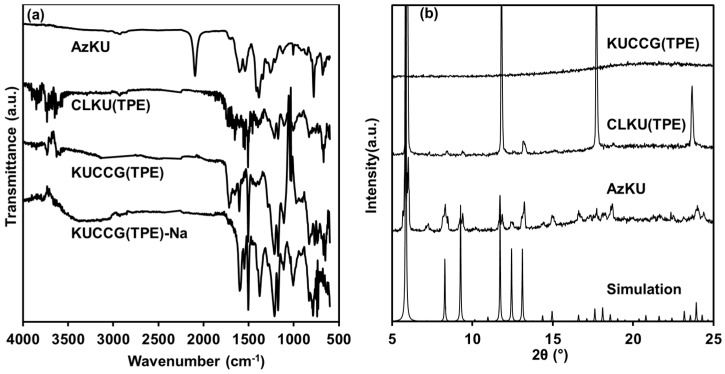
(**a**) Fourier transform infrared (FT-IR) spectra of AzKU, CLKU(TPE), KUCCG(TPE), and KUCCG(TPE)-Na; (**b**) XRD patterns of AzKU, CLKU(TPE), and KUCCG(TPE), and simulated pattern of KUMOF from single crystal data.

The synthesis of CCG was carried out by using KUMOF [30] and IRMOF-15 [31] as the parent metal organic framework (MOF) (Figure 1). At first, azide-functionalized KUMOF (AzKU) and IRMOF-15 (AzIR15) were synthesized by mixing Cu(II) and Azbpdc in a mixed solvent of *N*,*N*-diethylformamide (DEF) and DMSO for AzKU, or Zn(II) and Aztpdc in DEF for AzIR15 (Figure 1). The crosslinker TPE-CL4 was obtained via facile Williamson ether synthesis according to the literature process (Figure 1) [29]. The obtained rectangular crystal (AzKU) or cubic crystal (AzIR15) was then subjected to a crosslinking reaction withTPE-CL4 via Huisgen cyclization catalyzed by Cu(I) to afford CLKU(TPE) and CLIR15(TPE). After the crosslinking reaction of AzKU, the appearance was not changed; however, Fourier transform infrared (FT-IR) spectroscopy showed complete consumption of the peak at 2090 cm^−1^ attributed to azide stretching vibration (ν_N3_) (Figure 2a). On the other hand, FT-IR spectra of CLIR15(TPE) indicated the existence of persisting azide groups (Figure S1), probably due to the small pore aperture of IRMOF15 (12.3 Å) compared to that of KUMOF (24.7 Å). The X-ray diffraction (XRD) of AzKU and CLKU(TPE) indicated that the crosslinking reaction did not affect the crystallinity (Figure 2b). In a mixed hydrolyzing solution of conc. HCl/DMF (*v/v* = 1/5), CLKU(TPE) was hydrolyzed to yield KUCCG(TPE). The time-course observation of hydrolysis is shown in Figure 1d. From the outside of the green rectangular crystal, the hydrolyzing solution was penetrated with decoloration due to the extraction of Cu(II) ion from the secondary building unit (SBU) of KUMOF, while the rectangular shape and right angle remained, even after the hydrolysis. After 30 min immersion in the hydrolyzing solution, the green color completely faded, and FT-IR spectra of KUCCG(TPE) demonstrated the disappearance of carboxylate stretching vibration peak (ν_COO-_) at 1380 cm^−1^ observed for AzKU and CLKU(TPE) and the appearance of a carboxylic acid stretching vibration peak (ν_COOH_) at 1710 cm^−1^ (Figure 2a), indicating the successful hydrolysis of CLKU(TPE) to obtain KUCCG(TPE). The KUCCG(TPE) showed no diffraction pattern, indicating its amorphous nature (Figure 2b). Hydrolyzing CLIR15(TPE) resulted in the production of a spherical gel (Figure S2); thus, it lost its original polyhedral shape upon hydrolysis. The insufficient crosslinking density derived from a lack of crosslinker caused the result, due to the small aperture of IRMOF15.

**Figure 3:**
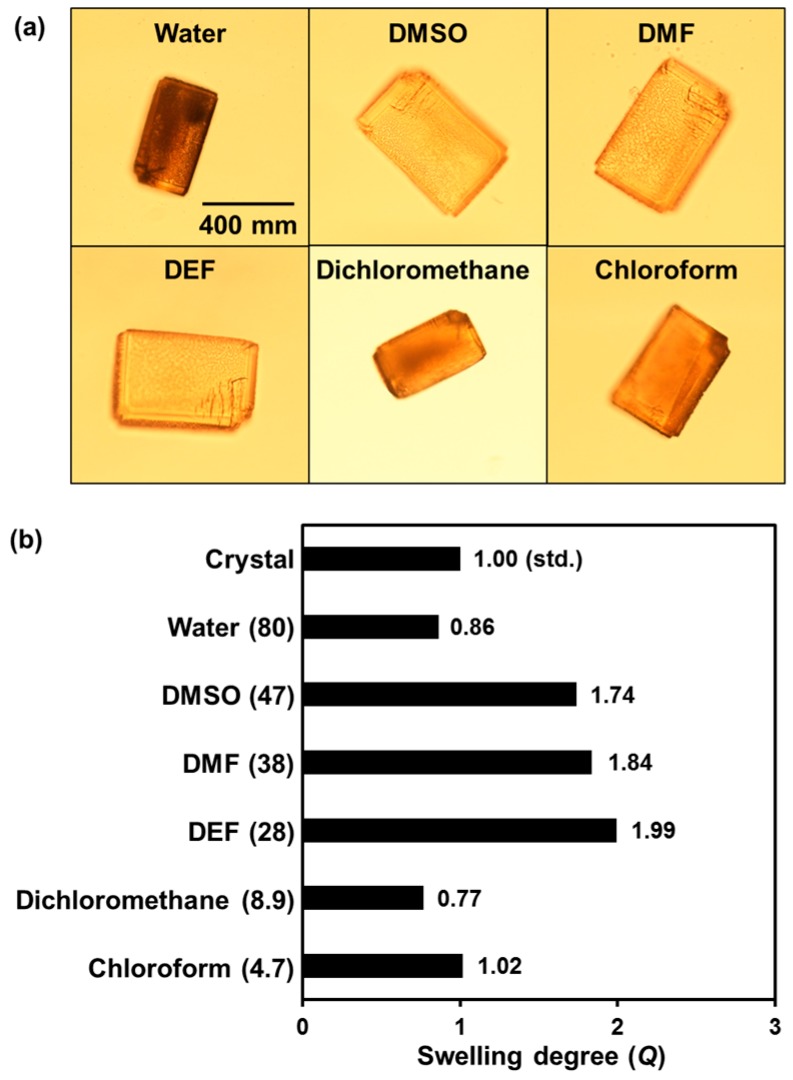
(**a**) Photographs of KUCCG(TPE) in various solvents; (**b**) Swelling degree (*Q*) of KUCCG(TPE) in various solvents. The value in parenthesis indicates the permittivity (ε) of each solvents. DEF: *N*,*N*-diethylformamide.

The swelling degree of obtained KUCCG(TPE) was investigated in various solvents, as shown in Figure 3. The equilibrium swelling degrees (*Q*) of the cubic gels were defined by the following equation:

$$Q=(L_{\mathrm{wet}}/L_{\mathrm{cryst}}{)}^{3}$$

where *L*_wet_ and *L*_cryst_ are average lengths of one side of CCG in wet and crystalline (before hydrolysis) state, respectively.

In aprotic polar solvents such as DMSO, DMF, or DEF, KUCCG(TPE) showed high *Q* value (e.g., 1.74 in DMSO, 1.84 in DMF, and 1.99 in DEF) due to suppression of hydrogen bonds among the carboxylic acid groups and good compatibility of phenyl rings of the organic ligands and crosslinkers. In other solvents, such as chloroform, dichloromethane, and water, KUCCG(TPE) was collapsed, and the *Q* values were found to be around 1:0.86 in water, 0.77 in dichloromethane, and 1.02 in chloroform. Therefore, the *Q* values depend on the compatibility between KUCCG(TPE) and the solvents, rather than the permittivity.

**Figure 4:**
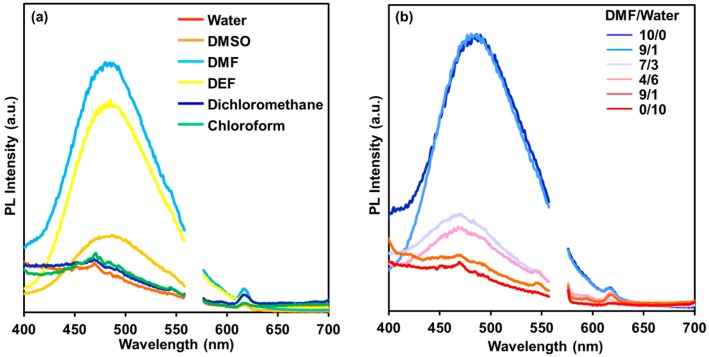
(**a**) Photoluminescence (PL) spectra of KUCCG(TPE) in various solvents (λ_ex_ = 280 nm); (**b**) Photoluminescence spectra of KUCCG(TPE) in mixed solvents of DMF and water (10/0 → 0/10, λ_ex_ = 280 nm). The spectra between 555 and 575 nm are omitted due to scattering light derived from the excitation light.

The photoluminescence property of KUCCG(TPE) was then investigated after immersing it in various solvents (Figure 4a). The excitation wavelength (280 nm) was selected to mainly excite the biphenyl moiety in the organic ligand. In a swollen state in good solvents such as DMSO, DMF, or DEF, KUCCG(TPE) showed a strong emission, mainly derived from the crosslinking TPE moiety at around 470–480 nm, indicating efficient energy transfer from biphenyl to TPE. On the other hand, in poor solvents, the emission from TPE moiety was largely suppressed. In other words, the photoluminescence intensity is governed by the swelling degree of the surrounding network polymer, due to the increase of scattering in the collapsed state of KUCCG(TPE), as can also be seen in Figure 3a. The measurement of absolute photoluminescence quantum yield (Φ_F_) reflected the photoluminescence spectroscopy data; thus, higher Φ_F_ was observed in DMF (3.0%, Table 1), and lower Φ_F_ was observed in water (0.7%). In our previous study, emission from TPE crosslinker in a conventional gel or elastomer was decreased in the swollen state in good solvents due to intramolecular motion of the TPE moiety [20,21,22]; however, in CCG, the confined and organized nature of the crystal crosslinked network polymer should be enough to achieve the RIM process, even in the swollen state.

To obtain deep insight into the swelling degree-dependent emission property, KUCCG(TPE) was immersed in mixed solvent of DMF and water with various ratios (*v/v* = 0/10–10/0, Figure 4b). Analogously to the above experiment, KUCCG(TPE) in swollen state in pure DMF showed large emission from the TPE moiety. An increase of water content strikingly diminished the emission from the TPE moiety, accompanying the decrease of swelling degree (1.84 to 0.86). This behavior is also derived from the increase of scattering in the collapsed state of KUCCG(TPE). From this point of view, a further increase of photoluminescence intensity was attempted via neutralization of carboxylic acid by 1 M NaOH aq. to obtain polyelectrolyte type CCG (KUCCG(TPE)-Na) having sodium carboxylate groups inside. After the neutralization, the FT-IR signal of ν_COOH_ at 1710 cm^−1^ disappeared, the swelling degree in water was improved to 3.17, and Φ_F_ also increased to 8.4% (Table 1).

The addition of a common salt experiment was carried out by immersion of KUCCG(TPE)-Na in 5 M NaCl aq., and upon the increase of salt concentration, the photoluminescence intensity was decreased in the common salt solution (Figure S3). These data suggested that the neutralization of carboxylic acid group provided an efficient improvement of photoluminescence intensity by increasing the swelling degree.

**Figure 5:**
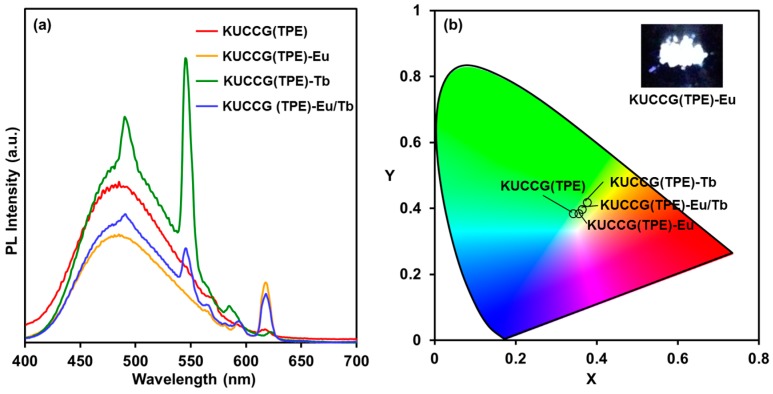
(**a**) Photoluminescence spectra of KUCCG(TPE), KUCCG(TPE)-Eu, KUCCG(TPE)-Tb, and KUCCG(TPE)-Eu/Tb (λ_ex_ = 280 nm); (**b**) CIE (Commission Internationale de L’Eclairage) 1931 (x,y) chromaticity diagram of KUCCG(TPE), KUCCG(TPE)-Eu, KUCCG(TPE)-Tb, and KUCCG(TPE)-Eu/Tb from the fluorescence spectra.

In order to finely control the emission color, we attested the introduction of photoluminescent rare earth ions to KUCCG(TPE)-Na. Because of the abundant carboxylate groups in KUCCG(TPE)-Na, it should be able to capture the added metal ions via coordination bonding. To prove this idea, KUCCG(TPE)-Na was immersed in 50 mM Eu(III) or Tb(III) aqueous solution to replace the metal ion. Additionally, a mixed metal ion solution of Eu(III) and Tb(III) (1/1, mol) was employed to tune the emission color. As a result, the obtained polymer gel showed a mixed photoluminescence derived from TPE moiety and the respective metal ion (Figure 5). The emission maximum of the TPE moiety was observed at around 480 nm, while that of rare earth ions was 620 nm for Eu(III), and 492 and 546 nm for Tb(III). This result indicated a balanced energy transfer from the biphenyl moiety to the TPE moiety and the rare earth ion. Both emission from Eu(III) and Tb(III) were exhibited for KUCCG(TPE)-Eu/Tb. Thus, the emission color was readily controlled by the kind of rare earth ion or the composition, as revealed by the illustration in the CIE diagram in Figure 5b.

## 4. Conclusions

In this article, we synthesized crystal crosslinked gels (CCGs) with AIE active crosslinker having a tetraphenylethene (TPE) moiety via crystal crosslinking (CC). Thus, the CCG was prepared from a metal-organic framework (MOF) with large pore aperture to allow the penetration of TPE crosslinker. As a result, a rectangular shaped CCG was obtained, which was derived from the original MOF. The obtained CCG showed a stimuli-responsive photoluminescence behavior depending on the swelling degree. A stronger photoluminescence intensity was observed at higher swelling degree, which is an opposite result compared to past research. The variable photoluminescence mechanism should rely on light scattering; thus, more AIE dye is excited in more transparent CCG at high swelling degree. Furthermore, the emission color was readily tuned via the introduction of luminescent rare earth ions to form a coordination bonding with residual carboxylate inside the CCG.

## Figures and Tables

**Table 1:** Summary of photoluminescence quantum yield (Φ_F_) and swelling degree (*Q*).

Sample	Solvent	Φ_F_ (%)	*Q*
KUCCG(TPE)	water	0.7	0.86
	DMF	3.0	1.84
KUCCG(TPE)-Na	water	8.4	3.17
	DMF	14.8	3.64

## Supplementary Materials

The following are available online at www.mdpi.com/2073-4360/9/1/19/s1. Figure S1: FT-IR spectra of AzIR15 and CLIR15(TPE). Figure S2: Time-course observation upon hydrolysis of CLIR15(TPE). Figure S3: Photoluminescence spectra of KUCCG(TPE) immersed in 5 M NaCl aq.
