# Supplementary material for: Crystal Crosslinked Gels with Aggregation-Induced Emissive Crosslinker Exhibiting Swelling Degree-Dependent Photoluminescence
Source: Polymers (Basel). 2017 Jan 6;9(1):19. doi: 10.3390/polym9010019 (PMC6432286; doi:10.3390/polym9010019)
Supplement: Supplementary file 1 [file polymers-09-00019-s001.pdf]

Supplementary Materials: Crystal Crosslinked Gels with Aggregation-Induced Emissive Crosslinker Exhibiting Swelling Degree-Dependent Photoluminescence

Tsuyoshi Oura, Ryosuke Taniguchi, Kenta Kokado and Kazuki Sada

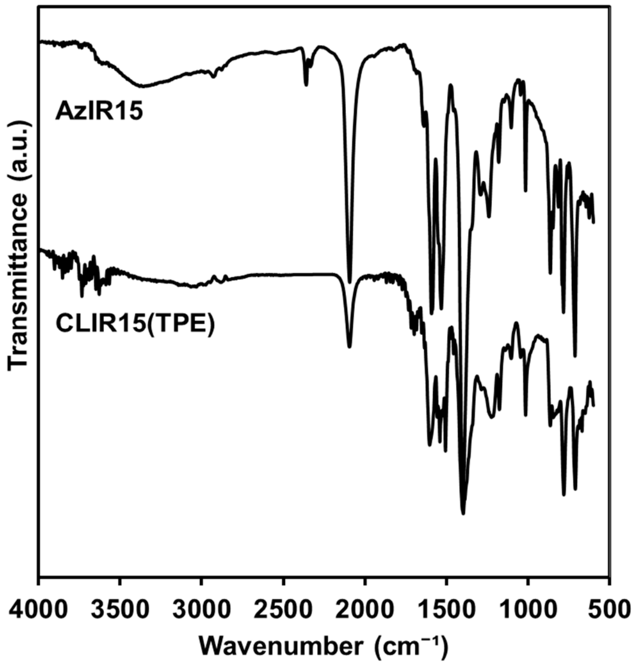

Figure S1. FT-IR spectra of AzIR15 and CLIR15(TPE).

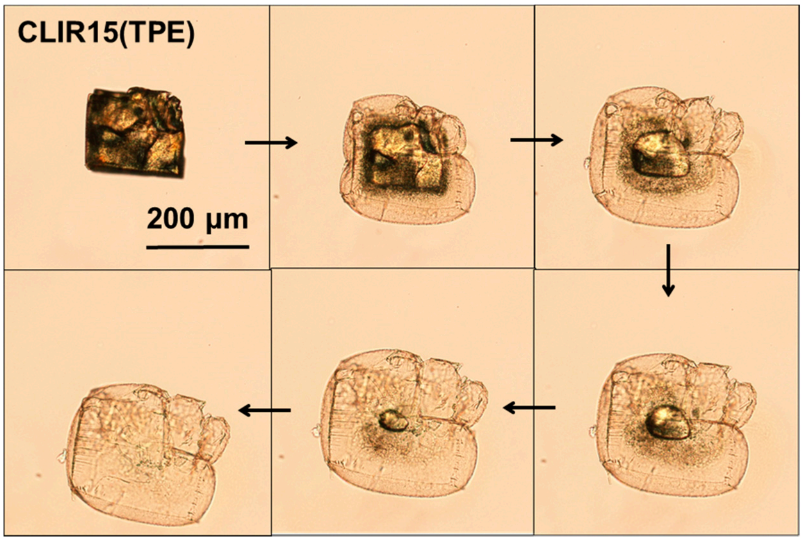

Figure S2. Time-course observation upon hydrolysis of CLIR15(TPE) in conc. HCl/DMF = 1/5 (*v*/*v*).

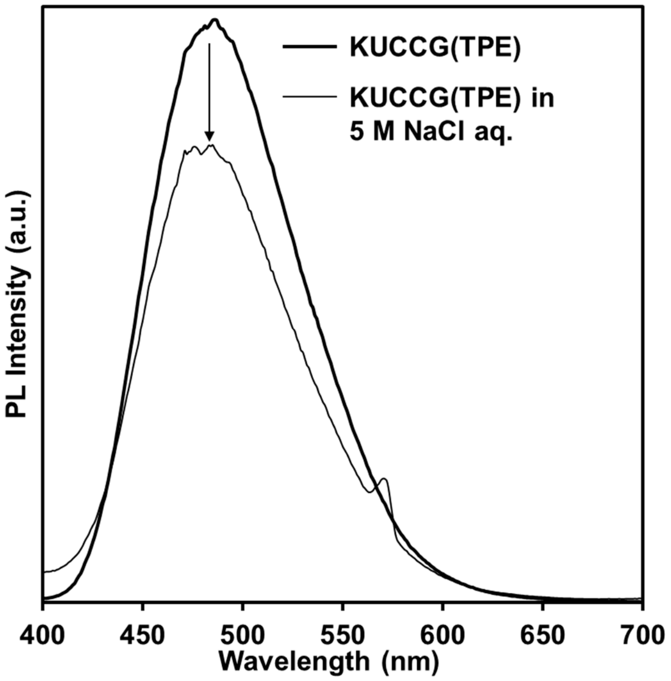

Figure S3. Photoluminescence spectra of KUCCG(TPE) before and after immersing in 5 M NaCl aq. (λ_{ex} = 280 nm).
